# Phototherapeutic Keratectomy for Concrete-Induced Corneal Chemical Injury: A Case Report

**DOI:** 10.7759/cureus.84604

**Published:** 2025-05-22

**Authors:** Hideki Fukuoka, Koji Kitazawa, Hiroto Yuge, Kaori Matsumoto, Chie Sotozono

**Affiliations:** 1 Department of Ophthalmology, Kyoto Prefectural University of Medicine, Kyoto, JPN

**Keywords:** alkaline injury, chemical injury, concrete, cornea, excimer laser, kinoshita classification, phototherapeutic keratectomy, ptk

## Abstract

Chemical injuries to the cornea, particularly those caused by alkaline substances like concrete, can lead to severe ocular damage and vision impairment. To the best of our knowledge, there are no published reports on the use of phototherapeutic keratectomy (PTK) specifically for concrete-induced corneal injuries. This case report presents the first documented successful management of a concrete-induced corneal chemical injury using PTK. A 69-year-old male patient presented with bilateral ocular injuries following exposure to concrete after a machine explosion at work. The right eye exhibited substantial damage, categorically designated as Grade 3b according to the Kinoshita classification system, manifesting as extensive limbal epithelial deficiency and the presence of concrete particles embedded within the corneal surface. Following an initial management plan that incorporated irrigation and anti-inflammatory therapy, on day 12 post-injury, the patient underwent PTK to address the superficial corneal opacification caused by concrete particles. Visual acuity exhibited a marked enhancement, progressing from 20/133 (uncorrected) preoperatively to 20/20 (with correction) postoperatively, accompanied by substantial clearance of the corneal opacity. This novel case demonstrates that phototherapy (PTK) is an effective treatment modality for corneal injuries caused by exposure to concrete, where superficial opacification and embedded particles pose a challenge to conventional management. The precise ablation capability of the excimer laser allows for targeted removal of concrete deposits without excessive damage to surrounding tissue, facilitating visual rehabilitation. This approach has the potential to establish a novel standard of care for the management of persistent corneal opacities resulting from concrete injuries.

## Introduction

Chemical injuries to the eye constitute ophthalmic emergencies that necessitate prompt intervention to mitigate permanent damage. Alkali injuries, such as those caused by concrete, are particularly destructive due to the high pH (12-13) of cement, which causes saponification of cell membranes, allowing deep penetration into the ocular surface [1]. Concrete injuries present a triple threat: alkaline damage from cement, thermal injury from the exothermic hydration reaction, and mechanical trauma from embedded particles (sand and gravel) [2,3].

The management of such injuries is intricate, with initial treatment focusing on immediate irrigation to normalize pH, followed by anti-inflammatory therapy and management of complications [4]. However, conventional treatment may prove ineffective when concrete particles become embedded in the corneal surface, resulting in persistent opacification and reduced visual acuity. In severe cases, permanent visual impairment may result despite aggressive conventional therapy.

Phototherapeutic keratectomy (PTK) is a surgical procedure that utilizes an excimer laser to precisely ablate superficial corneal tissue. Initially developed for the treatment of corneal dystrophies and degenerations, PTK has emerged as a valuable option for treating superficial corneal opacities and irregularities [5]. The 193-nm wavelength of the excimer laser has been demonstrated to break molecular bonds with minimal thermal effect on surrounding tissues, allowing for precise removal of opacified tissue and foreign materials [6].

Despite the documented use of PTK for various corneal pathologies, a thorough literature search revealed no prior reports specifically addressing its application in concrete-induced corneal opacities. This finding signifies a notable lacuna in the extant clinical knowledge, as concrete is a prevalent industrial material, and ocular injuries due to this material are not infrequent in occupational settings.

This case report documents the initial successful application of PTK in the management of a severe, concrete-induced corneal chemical injury, emphasizing its efficacy in removing embedded concrete particles and enhancing visual outcomes. In light of the distinctive challenges posed by concrete particles embedded in corneal tissue, this innovative application of PTK has the potential to offer valuable guidance to ophthalmologists in the future when managing cases of a similar nature.

## Case presentation

**Figure 1:**
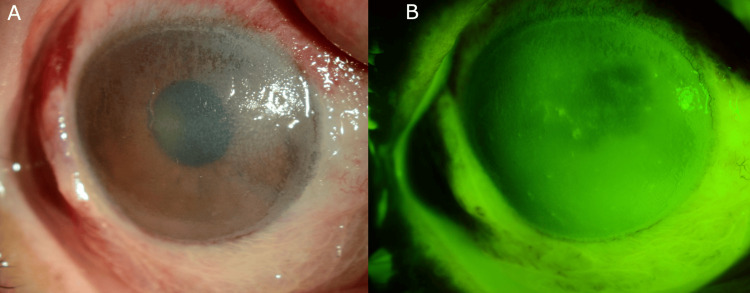
Clinical photographs of concrete-induced corneal chemical injury (A) Initial presentation of the right eye showing severe corneal damage with extensive conjunctival congestion and grayish concrete deposits presumably embedded in Bowman’s layer. Note the diffuse corneal opacity and complete epithelial defect consistent with Grade 3b chemical injury according to the Kinoshita classification. (B) Fluorescein staining highlighting the extent of the epithelial defect. The yellow staining indicates corneal and conjunctival epithelial damage, with the concrete particles in Bowman’s layer.

A 69-year-old male with no significant past medical history presented to the ophthalmology department following an occupational accident in which a concrete machine exploded, exposing both eyes to concrete splashes. The patient had initially been seen at a local clinic, where his eyes were irrigated with 900 ml of saline. However, due to the severity of the injury and the inability to remove the concrete particles, he was referred to our institution. Upon arrival, an additional 2,000 ml of saline irrigation was performed bilaterally. A preliminary evaluation of the patient’s visual acuity revealed a best-corrected visual acuity (BCVA) of 20/133 in the right eye and 20/50 in the left eye. A slit-lamp examination of the right eye revealed significant conjunctival congestion, concrete deposits on the cornea (Figure 1A, 1B), and a total corneal and conjunctival epithelial defect accompanied by presumed complete loss of limbal palisades of Vogt. This finding is consistent with a Grade 3b chemical injury, as classified by the Kinoshita system (Table 1) [7], which has been demonstrated to be a simple yet reliable predictor of visual prognosis in chemical and thermal injuries [8]. The left eye presented with only a partial corneal epithelial defect, classified as Grade 2, and healed for a few days.

**Table 1:** Kinoshita classification (severity classification of acute chemical burns POV refers to “palisades of Vogt” (limbal palisades).

Grade	Clinical findings	Severity
Grade 1	Conjunctival hyperemia (+), corneal epithelial defect (-)	Mild
Grade 2	Conjunctival hyperemia (+), partial corneal epithelial defect (+)	Moderate
Grade 3a	Complete corneal epithelial defect (+), presence of POV (+)	
Grade 3b	Complete corneal epithelial defect (+), complete loss of POV	Severe
Grade 4	Necrosis of more than 50% of the limbal conjunctiva, complete corneal epithelial defect, complete loss of POV	

**Figure 2:**
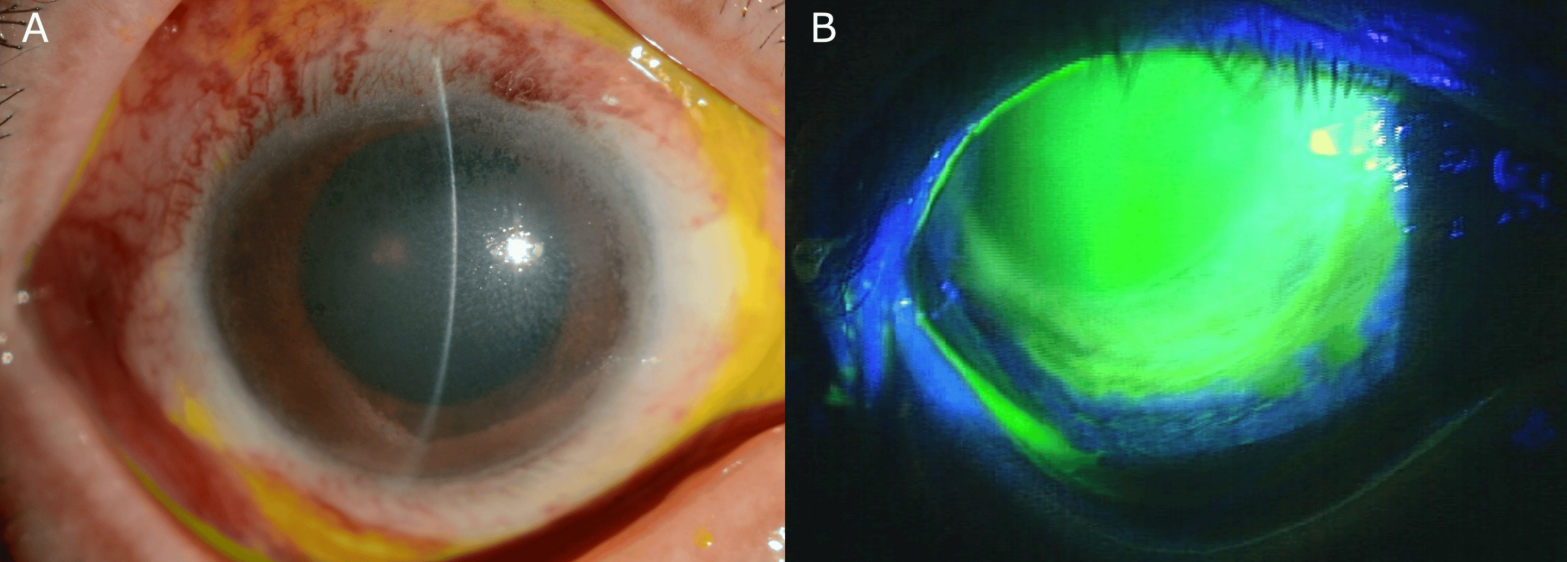
Clinical photographs of the right eye before PTK (A) Slit-lamp photograph showing persistent corneal opacification with grayish discoloration of the cornea due to embedded concrete particles. Note the significant conjunctival hyperemia, indicating an ongoing inflammatory response. An SCL is visible on the corneal surface. (B) Fluorescein staining demonstrating persistent epithelial defect and irregular corneal surface. The fluorescence highlights areas of compromised epithelial integrity. The image shows extensive involvement of the total cornea, explaining the patient’s reduced visual acuity (20/133) prior to PTK intervention. PTK, phototherapeutic keratectomy; SCL, soft contact lens

The presence of concrete deposits on the corneal surface introduced a significant challenge to the accuracy of the assessment. The patient was admitted to the hospital for intensive treatment, which included the following medications: a single intravenous dose of methylprednisolone (125 mg), oral cyclosporine (50 mg, three times daily), oral prednisolone (10 mg, daily), and topical antibiotics and anti-inflammatory agents (levofloxacin, atropine, and betamethasone). Following an initial evaluation, the patient was fitted with a soft contact lens for the right eye. This procedure was designed to promote epithelial healing. Although conjunctival epithelialization was observed, the corneal surface demonstrated only minimal improvement. The BCVA improved to 20/50 (with correction), but the corneal opacity due to concrete deposits persisted (Figure 2A, 2B).

Due to the persistent corneal opacification from embedded concrete, PTK was planned and subsequently performed on day 12 post-injury using the EC-5000 (NIDEK Co., Ltd., Gamagori, Japan) (Video 1). This timing was crucial, as it represented the period before the cornea could be covered by conjunctival epithelium through conservative treatment alone. Prior to deciding on PTK, we attempted removal using surgical sponges and a golf knife, but these conventional methods proved ineffective against the firmly embedded concrete particles. The PTK procedure entailed the initial ablation of 30 μm of corneal tissue, employing an 8-mm optical zone and a 9-mm transition zone. This was followed by a subsequent ablation of 30 μm, utilizing a 6-mm optical zone and a 7-mm transition zone. This two-staged approach was adopted because the optimal ablation depth was unclear, allowing us to treat both the optical zone and peripheral cornea while minimizing impact on the limbal area. Subsequent to the procedure, a therapeutic contact lens was placed.

**Video 1:** PTK procedure for concrete-induced corneal chemical injury PTK, phototherapeutic keratectomy

**Figure 3:**
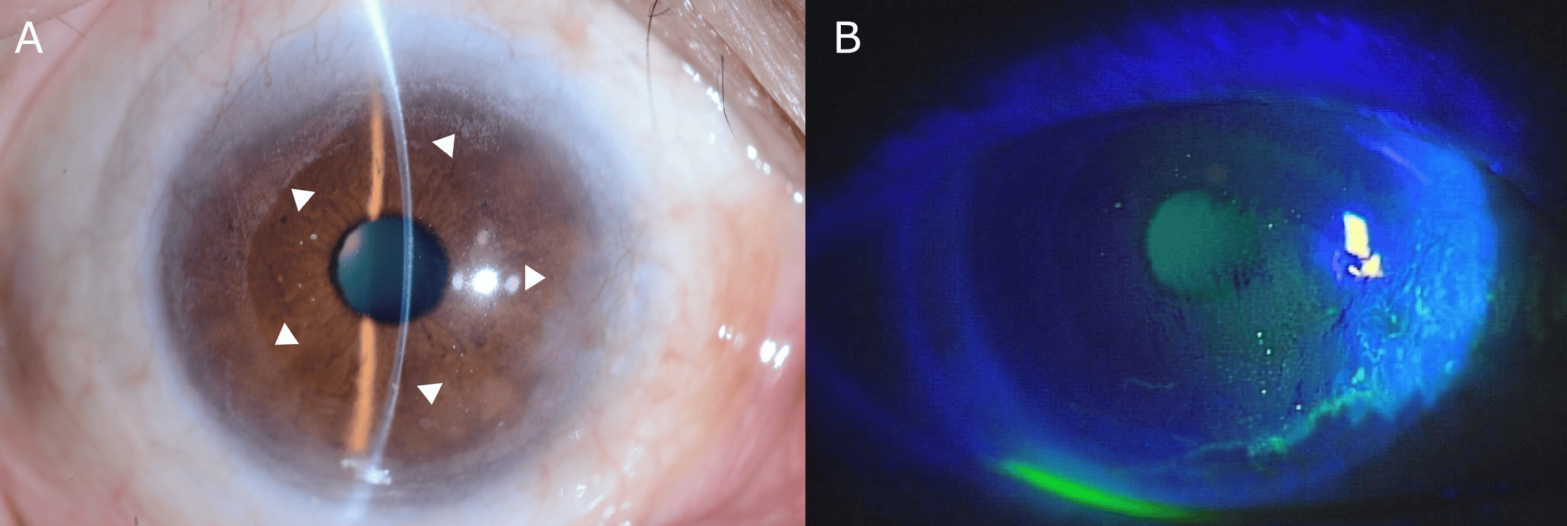
Long-term follow-up at three years post-injury (A) This slit-lamp photograph of the right eye shows significant improvement in corneal clarity three years after injury and PTK. The concrete deposits have been successfully removed, resulting in a smooth corneal surface with minimal residual peripheral opacity (indicated by the white arrows). The iris details are clearly visible, indicating substantially improved corneal transparency. (B) Fluorescein staining demonstrating no epithelial defect, confirming successful healing after PTK. PTK, phototherapeutic keratectomy

The procedure targeted the corneal areas with concrete deposits, using the excimer laser's precise ablation capabilities to remove the superficial corneal layer containing the concrete particles. After PTK, the patient exhibited a significant recovery. The treated area exhibited substantial clearance of the concrete-induced opacity. The BCVA demonstrated an initial improvement to 20/40 and further improved to 20/20 during follow-up, with this excellent visual outcome maintained throughout the three-year follow-up period (Figure 3A, 3B).

## Discussion

This case demonstrates the efficacy of PTK in managing concrete-induced corneal chemical injuries, particularly in addressing persistent opacification caused by embedded concrete particles. To the best of our knowledge, this report is the first to utilize PTK specifically for concrete-related corneal injuries, thus demonstrating a novel therapeutic approach for these challenging cases.

Our comprehensive literature search included not only major indexed databases (PubMed, Embase, Scopus, and Google Scholar) but also extended to conference proceedings, non-indexed journals, and gray literature through May 10, 2025, confirming that this case represents the first documented instance of PTK for concrete-induced corneal chemical injury. This systematic search, employing a combination of terms including “phototherapeutic keratectomy”, “PTK”, “concrete”, “cement”, “chemical injury”, and “corneal burn”, confirmed that our case represents the first documented instance of this specific therapeutic approach for managing corneal opacities caused by embedded concrete particles.

Concrete injuries pose a distinctive challenge in the field of ophthalmology due to their intricate nature, which encompasses a combination of alkaline damage (pH 12-13), thermal injury from exothermic hydration reactions, and mechanical trauma from particulate matter [9]. The composition of concrete - approximately 21% cement, 34% sand, 34% gravel, and 11% water - results in multifaceted corneal damage that conventional treatments struggle to address completely.

The initial management of chemical injuries is focused on copious irrigation, anti-inflammatory therapy, and promoting epithelial healing [10]. However, in cases involving the presence of concrete particles within the cornea, conventional treatment regimens may prove inadequate in restoring optimal corneal clarity and visual function. This case exemplifies PTK’s efficacy in addressing such scenarios.

The excimer laser utilized in PTK functions at a wavelength of 193 nm, enabling precise cleavage of molecular bonds through a process known as photoablation without significant heat generation [11]. This property is particularly valuable in removing embedded concrete particles without causing additional thermal damage to the already compromised cornea. In contrast to mechanical debridement or superficial keratectomy, which have the potential to inflict further trauma to the injured cornea, the excimer laser has the capacity to remove microscopic layers of tissue with submicron precision, rendering it ideal for targeting concrete deposits.

In this case, the efficacy of PTK is substantiated by the marked enhancement in visual acuity, from 20/133 preoperatively to 20/20 (with correction) postoperatively and following a healing period. This substantial enhancement underscores PTK’s capacity to efficiently eradicate concrete-induced opacities that demonstrate resistance to conventional treatment methodologies.

The therapeutic mechanism of PTK in concrete injuries likely involves several factors. The process of direct removal of superficial concrete particles (cement, sand, and gravel) embedded in Bowman’s layer is a procedure that should be performed with the utmost caution and precision. The elimination of opacified corneal tissue, damaged by the alkaline and thermal effects of concrete, is a critical step in the treatment process. The creation of a smoother corneal surface has been demonstrated to reduce light scatter and improve optical quality. The elimination of microscopic foreign bodies is crucial for preventing persistent inflammation and facilitating normal healing processes that would otherwise be impeded by the presence of these particles. It is imperative to exercise caution and avoid laser ablation of the limbal area during PTK, as this procedure could potentially exacerbate limbal stem cell deficiency in eyes that are already compromised by chemical injuries.

It is imperative to acknowledge the limitations and potential complications associated with PTK. Refractive changes are a common occurrence following PTK, typically inducing hyperopic shifts proportional to the depth of ablation [12]. According to extant literature, an approximate 3 diopters of hyperopic shift may occur with 120-130 μm of corneal ablation. In this case, a significant refractive error was observed postoperatively, necessitating correction with contact lenses. It is important to note that other potential complications may include corneal haze, delayed epithelial healing, and infection. However, these were not observed in the present case.

The positive outcome in this case indicates that PTK should be considered earlier in the treatment algorithm for concrete-related corneal injuries, particularly when opacification persists despite conventional management. This approach may help prevent chronic complications and expedite visual rehabilitation.

## Conclusions

This case report is noteworthy for its contribution to the existing body of literature on the subject, as it is the first to demonstrate the efficacy of PTK as a treatment modality for concrete-induced corneal chemical injuries. The report highlights the utility of PTK, particularly in cases where conventional treatment methods prove ineffective in addressing embedded particles and the subsequent opacification. The precise ablation capabilities of the excimer laser allow for targeted removal of concrete deposits without excessive damage to surrounding tissue, facilitating visual rehabilitation. The distinctive characteristics of concrete injuries, which encompass a combination of alkaline damage, thermal injury, and persistent particulate matter, render them particularly challenging to manage with conventional approaches alone. PTK offers a novel solution by addressing the mechanical component of these injuries with minimal additional trauma to the already compromised cornea. The marked enhancement in visual acuity exhibited by the patient (from 20/133 to 20/20 with correction) serves to underscore the efficacy of the aforementioned approach. For ophthalmologists confronted with severe chemical injuries caused by concrete, PTK should be considered as a valuable option when persistent opacification and embedded particles are present. It is hereby proposed that this approach be incorporated into treatment algorithms for concrete-related corneal injuries that do not respond adequately to conventional management.

Further research is necessary to establish standardized protocols for the timing and techniques of PTK in concrete-related corneal injuries. Such research should include larger case series. Moreover, comparative studies evaluating PTK against other surgical interventions for concrete injuries would facilitate the refinement of treatment guidelines. Given the industrial prevalence of concrete and the sight-threatening nature of these injuries, such research would have a significant clinical impact.
